# Redefining Neck Rejuvenation: The Novel Hyoid-to-Mastoid Crevasse Neo-Ligament and OnderKaak Angle in Deep Plane Neck Lift

**DOI:** 10.1093/asjof/ojag009

**Published:** 2026-01-20

**Authors:** R Brannon Claytor, Patricia M Fuentes, Grace Tolan

## Abstract

**Background:**

Neck rejuvenation has evolved from superficial skin tightening to deeper structural modifications targeting the platysma, submental and subplatysmal fat, and the submandibular gland (SMG). Significant controversy surrounds the surgical maneuvers necessary to achieve the ideal neck.

**Objectives:**

The objective is to introduce the Hyoid-to-Mastoid Crevasse (HMC) neo-ligament for neck rejuvenation that elevates the SMG via a suture secured from the hyoid periosteum to the mastoid crevasse. Moreover, we introduce the OnderKaak (OK) angle, representing the region overlying the submandibular gland, best appreciated from the posterior oblique view, also known as the reverse ogee view.

**Methods:**

Patients who underwent deep plane facelift and neck lift with HMC neo-ligament performed between January 2024 and October 2024 were included. Cervicomental (CMA), gonial (GA), and OK angle measurements were recorded pre and postoperatively. Patients were divided into two cohorts: without SMG resection (“no-SMG resection”) and with SMG resection (“SMG resection”).

**Results:**

A total of 25 patients were included. Fifteen patients (60%) underwent partial SMG resection, and 10 (40%) did not. Both groups demonstrated significant improvement in cervicomental, gonial, and OK angles (all *P* < .05). There was no difference in the mean change of CMA, GA, and OK angles between both groups (all *P* > .05).

**Conclusions:**

The HMC neo-ligament can be used to achieve improved neck aesthetics, thus offering a new technique for neck lift surgery. This technique may be used with and without submandibular gland resection. We also introduce the novel OnderKaak angle to better characterize the improved contours delivered by the HMC neo-ligament.

**Level of Evidence: 4 (Therapeutic):**

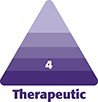

The aging neck results from multifactorial changes, commonly including platysmal laxity, redistribution of submental fat, and submandibular gland (SMG) ptosis. To address these cervical changes, plastic surgeons have developed techniques targeting both the superficial and deep layers of the neck.^1^

The elements of a youthful neck were first described by Ellenbogen, which included a distinct inferior mandibular border, a subhyoid depression, and a cervicomental angle measuring between 105 and 120 degrees.^2^ To achieve this criteria, Ellenbogen described complete dissection of the platysma muscle to create a platysma sling in addition to submental defatting. In 1992, Feldman introduced the corset platysmaplasty, utilizing midline and lateral sutures to plicate the platysma enhancing neck contour.^3^ However, this technique led to a midline ridge deformity. Later, in 1995, Giampapa^4^ presented the interlocking suspension suture spanning from mastoid to mastoid, but lacked sufficient SMG support, causing a “hammocking effect.” Techniques by Connell and Baker involved platysma flaps anchored to the mastoid fascia, which led to improved aesthetic results. However, these techniques inadequately provided sufficient support to the submandibular gland to effectively address prolapse.^5-7^ Talei's mastoid crevasse technique, for example, has led to more definition at the gonial angle, though optimizing the region overlying the submandibular glands continues to be a vexing problem.^5^ This is particularly important for patients who have substantial neck fullness or low SMG position, hindering them from achieving optimal neck contours.

To address this gap, the primary surgeon developed the Hyoid-to-Mastoid Crevasse (HMC) neo-ligament surgical technique that establishes a neo-ligament connecting the hyoid to the mastoid bone periosteum. This suture can be used in cases of submandibular gland preservation or partial resection.

Achieving the ideal neck has become a critical component of facial rejuvenation outcomes, thus greater attention has been placed on quantifying key angles such as the cervicomental (CMA) and gonial (GA) angles.^8^ Halani et al demonstrated this in an updated analysis of Ellenbogen's ideal neck, defining the ideal values to range from 90 to 120° for the CMA and 120 to 140° for the GA.^7-9^ However, these angles do not accurately evaluate the area directly under the mandible, leaving the region of the SMG unmeasured.

Complementing the HMC neo-ligament is the introduction of the OnderKaak (OK) angle, derived from the Dutch term “onderkaak,” meaning “lower jaw.” This term builds on the contributions of Jan F.S. Esser in maxillofacial surgery during World War I.^10^ The OK angle describes the neck region below the mandible directly over the SMG, best visualized from a posterior oblique or reverse ogee view. Its purpose is to provide a standardized and measurable aesthetic unit that reflects the improvements achieved by the deep plane neck lift HMC neo-ligament.

The objective of this study is to introduce the HMC neo-ligament, which is designed to vertically lift and support the submandibular glands and deep structures of the neck. Moreover, the OK angle is introduced to provide a conceptual framework to objectively evaluate the region overlying the submandibular gland.

## METHODS

### Study Design and Patients

This was a retrospective chart review of 25 patients who underwent deep plane facelift and neck lift surgery using the HMC neo-ligament technique between January 2024 and October 2024. The study cohort was divided into two groups: patients who did not undergo gland resection (“no-SMG resection”) and those who underwent partial submandibular gland resection (“SMG resection”). All procedures were performed by the primary surgeon under tumescent local anesthesia in a Class A QUAD-A surgical facility. Institutional review board approval was obtained by Main Line Health (E-25-5484). All patients provided written consent for the use of their photographs and videos.

### Study Variables

Demographic variables collected included age, body mass index (BMI), gender, comorbidities, and smoking status. Operative details included operative time (minutes) and additional procedures performed. Postoperative complications collected include seroma, neuropraxia, hematoma, neck tightness, sialocele, skin necrosis, and revision surgery.

Standardized 360° preoperative and postoperative imaging was obtained using the oVio360 system (oVio Technologies, Nappanee, IN). Angular measurements including the cervicomental angle (CMA), gonial angle (GA), and OnderKaak angle (OK) were measured in degrees using the Angle Meter 360 application (Alexey Kozlov, 2018). All measurements were performed by a single blinded evaluator to reduce interobserver variability. The images used for angle measurements were taken at the most recent follow-up visit available. OnderKaak angle head positioning is standardized by having patients stand on a marked area within the 360° oVio imaging device. The oVio 360° frame was kept consistent to obtain the OnderKaak angle image (Frame: #229).

### Statistical Analysis

Statistical analysis was performed using IBM SPSS Statistics version 30.0 (IBM Corporation, Armonk, NY). Descriptive statistics were reported for all variables. Categorical variables were compared between groups using the Pearson chi-square test or Fisher's exact test when appropriate. Continuous variables were compared using the Mann–Whitney U test. Preoperative and postoperative angle changes within groups were analyzed using the Wilcoxon signed-rank test, and intergroup comparisons were performed using the Mann–Whitney U test. Statistical significance was set at *P* < .05.

### Surgical Technique

Local tumescent anesthesia of 500 mL normal saline with 50 mL of 1% lidocaine with 1 cc of epinephrine (1:1000) was used. Beginning with a submental incision, the dissection is made anterior to the platysma muscle and deep to the subcutaneous fat. The central subplatysmal fat is resected to the level of the cricoid cartilage, and the platysma muscle is elevated to expose the anterior belly of the digastric muscles. Dissection continues inferiorly along the digastric muscle to the capsule of the submandibular gland. Subplatysmal fat is resected laterally to the level of the submandibular gland.

Preoperative determination regarding preservation or partial resection of the SMG is based on both clinical exam and patient preference. If the gland is found to be enlarged or prolapsed below the border of the mandibular bone, the recommendation is for SMG resection to the level of the mandibular margin. Some patients elect not to have their gland resected despite clinical findings of prominence. In these patients, the capsule is left undisturbed, but the dissection over the capsule underneath the platysma is carried out to provide mobilization of the platysma and access for the hyoid-to-mastoid suture.

If SMG partial resection is performed, it involves wide mobilization within the capsule, where any penetrating vessels are cauterized. The resection angle is based on the degree of prolapse below the mandible angle.

**Figure 1.**
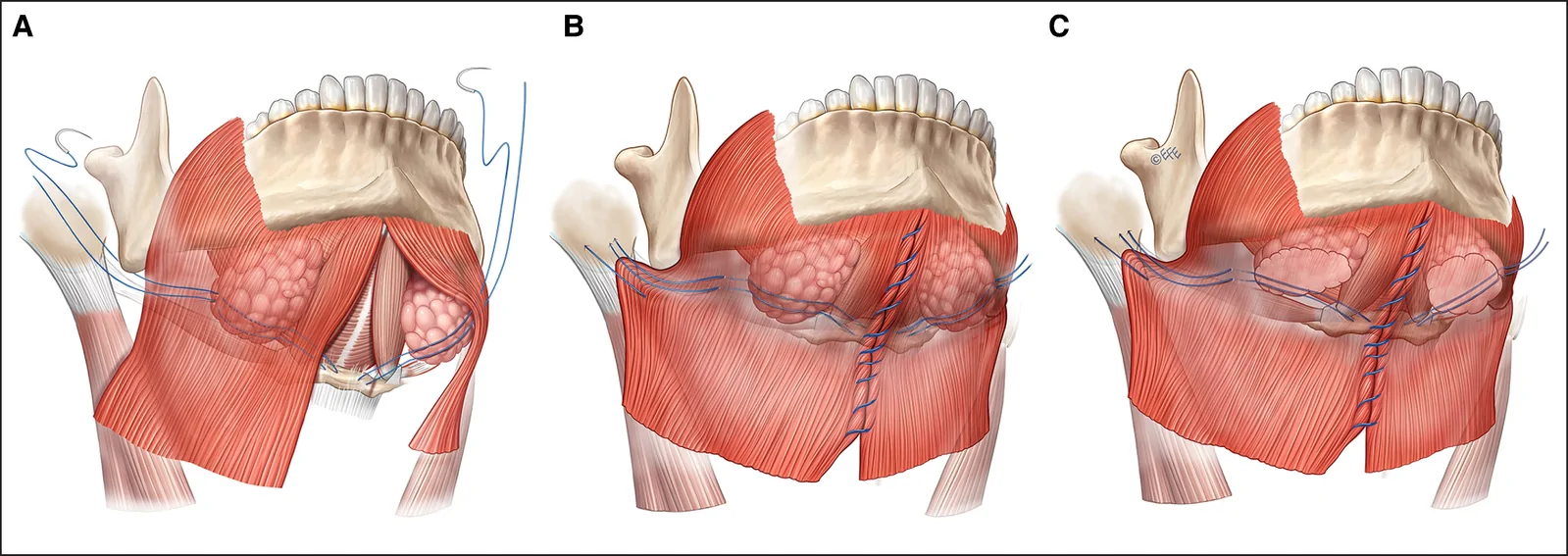
This figure shows an anterior view of the Hyoid-to-Mastoid Crevasse (HMC) neo-ligament technique. (A) A suture is anchored from the hyoid periosteum, through the underside of the platysma, to the mastoid crevasse forming a neo-ligament. (B) The HMC neo-ligament supports the submandibular glands. The central corset platysmaplasty is then performed through the submental incision. (C) In the case of partial SMG resection, the HMC neo-ligament technique provides direct pressure to the cut surface of the SMG. Illustrations provided by Dr. Efe Levante, CMI.

The neo-ligament is placed using a 3-0 PDS (Ethicon US, LLC, Raritan, NJ) suture beginning on the superficial surface of the platysma, through the platysma, and directly over the SMG. Then, it is passed through the hyoid periosteum (Figure 1). The exact position of the hyoid bone is determined by either instructing the patient to swallow or direct palpation of the bone, demonstrating the pivot point of the hyoid bone. The suture is then passed laterally directly over the SMG and through the platysma muscle. Both ends of the suture are passed laterally under the skin flap and temporarily secured with a hemostat (Video 1, available online at www.asjopenforum.com).

A 3-0 PDS suture is used to perform a platysmaplasty at the midline. Additional platysma support is provided with a 2-0 Prolene (Ethicon US, LLC, Raritan, NJ) suture. This suture is placed via the submental incision directly over the SMG into the platysma in a horizontal orientation as a single purchase. Then, it is passed laterally and secured with a hemostat.

The deep plane SMAS dissection is carried in a curvilinear orientation with elevation of the platysma along the jawline. At the level of the sternocleidomastoid muscle, the dissection is turned inferior and elevated along the sternocleidomastoid muscle for 4 cm. The platysma is elevated approximately 4 cm along the entire extent of incision.

**Figure 2.**
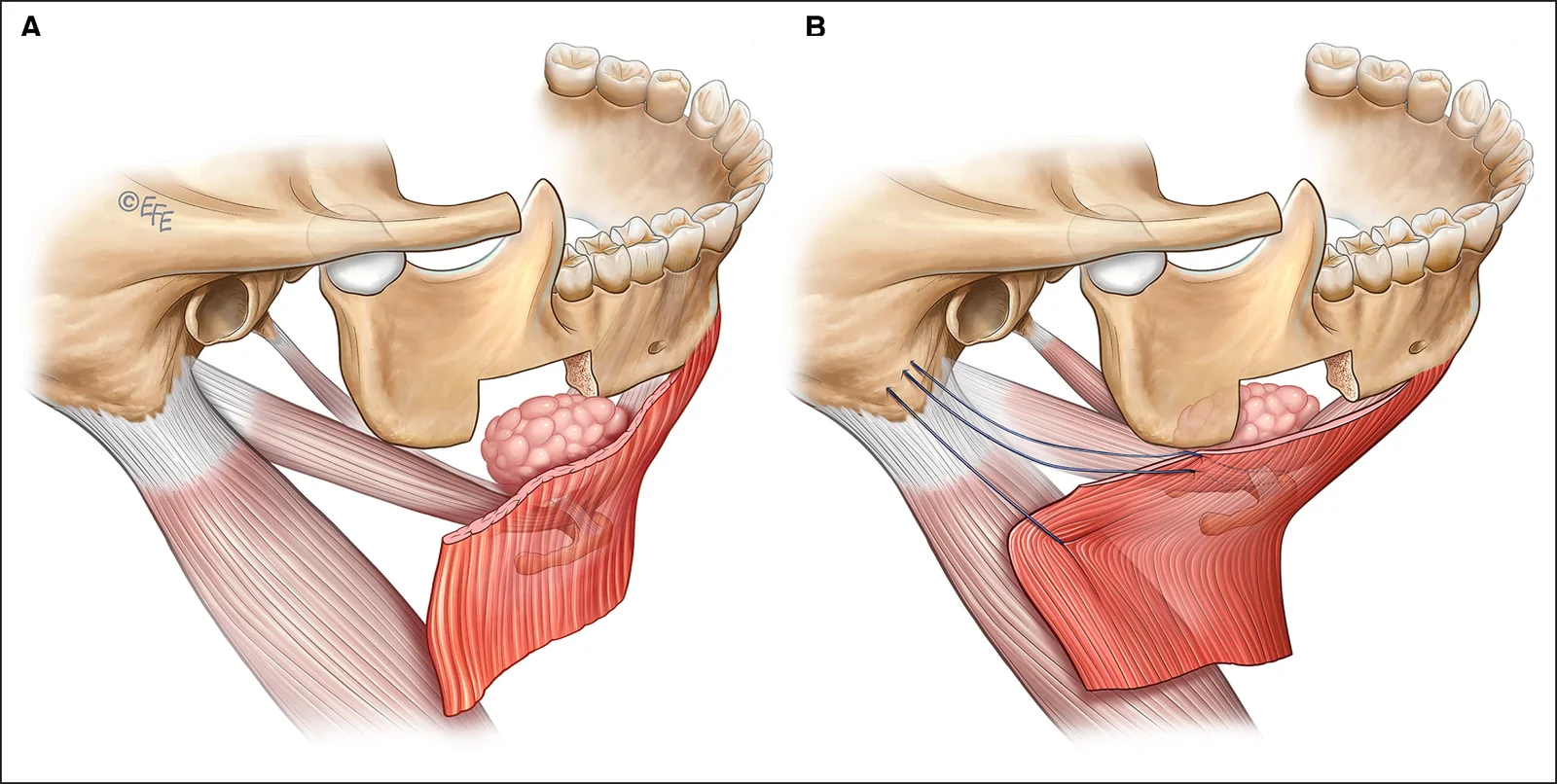
This figure shows a lateral view of the hyoid-to-mastoid neo-ligament technique. (A) Pre-operative illustration of the submandibular ptosis below the level of the angle of the mandible and platysmal laxity. (B) After intervention, the HMC neo-ligament vertically lifts the submandibular gland above the angle of the mandible and platysma to create a defined neck contour. Illustrations provided by Dr. Efe Levante, CMI.

As described by Talei, a lateral approach of the mastoid crevasse is developed by incising directly over the mastoid bone and through the fascia in a curvilinear fashion (Video 2, available online at www.asjopenforum.com).^11^ The mastoid crevasse is a pocket created at the mastoid region during deep plane neck lifting, which serves as a site for fixation and suspension of the platysma. The previously elevated lateral border of the platysma muscle is secured using a figure of eight 2-0 Prolene inset into the mastoid crevasse. The 2-0 Prolene previously placed over the SMG is secured into the mastoid crevasse. Finally, the 3-0 PDS previously anchored on the hyoid periosteum is secured into the mastoid crevasse creating the hyoid-to-mastoid crevasse neo-ligament (Figure 2).

### Postoperative Care

After the procedure, the patient's head is wrapped with Kerlix (KPR U.S., LLC, Dublin, OH) over Xeroform (DeRoyal Industries, Inc., Powell, TN, USA) gauze and ACE (3 M, Maplewood, MN) wrap with moderate pressure set by the primary surgeon. The patient is advised to keep the wrap for 24 hours until reevaluated by the primary surgeon.

## RESULTS

A total of 25 patients who underwent deep neck lift with the hyoid-to-mastoid crevasse were included (24 females and 1 male). Patients were divided into two groups: those who did not undergo submandibular gland (SMG) resection (“no-SMG resection”) (n = 10, 40%) and those who underwent partial SMG resection (“SMG resection”) (n = 15, 60%). SMG resection was performed in patients with clinically evident gland descent below the mandibular border on preoperative physical examination and confirmed intraoperatively.

Table 1 summarizes all demographics, operative variables, and postoperative complications. Angle measurements were taken on average 215 days postoperatively (range: 16-576 days). There were no differences in baseline characteristics between the two groups. The mean age was 64.2 ± 6.5 years (range, 57-79) in the no-SMG resection group and 63.4 ± 5.5 years (range, 55-71) in the SMG resection group (*P* = .946). The mean BMI was 24.8 ± 4.5 kg/m² (range, 19.6-33.3) in the no-SMG resection group, and 25.5 ± 4.2 kg/m² (range, 19.4-36.3) in the SMG resection group (*P* = .567). The prevalence of comorbidities such as hypertension, hyperlipidemia, autoimmune disorders, and diabetes mellitus was similar between groups (all *P* > .05). The mean operative time for the no-SMG resection group was 264.3 ± 56.8 minutes, and 262.3 ± 52.8 minutes for the SMG resection (*P* = .924).

**Table 1.** Demographic, Operative Characteristics, and Postoperative Outcomes of the Study Cohort

Variable, n (%) or mean ± SD	No SMG resection	SMG resection	*P*-value
Total patients	10	15	—
Age (years)	64.20 ± 6.47 (range: 57 - 79)	63.40 ± 5.50 (range: 55 - 71)	.946
Sex, n (%)			.405
Female	10 (100%)	14 (93.3%)	
Male	0	1 (6.6%)	
BMI (kg/m²)	24.80 ± 4.47 (range: 19.6 - 33.3)	25.45 ± 4.23 (range: 19.39 - 36.31)	.567
Comorbidities, n (%)			
Any comorbidity	7 (70%)	9 (60%)	.691
Hypertension	2 (20%)	1 (6.7%)	.543
Diabetes mellitus	1 (10%)	0	.400
Hyperlipidemia	1 (10%)	3 (20%)	.626
Scarring or keloids	0	1 (6.7%)	.306
DVT or hypercoagulability	1 (10%)	0	.400
Bleeding	0	1 (6.7%)	.326
Autoimmune disorder	3 (30%)	3 (20%)	.653
Operative characteristics			
Procedure length (minutes)	264.3 ± 56.83 (range: 199-378)	262.33 ± 52.82 (range: 197-370)	.924
Additional procedures	4 (40%)	8 (53.3%)	.688
Blepharoplasty	4	3	
Abdominal liposuction	—	1	
LaMiNa^a^	—	1	
Browlift	—	2	
Facial fat grafting	—	1	
Postoperative complications			
Any complication	3 (30%)	4 (26.7%)	.856
Seroma	—	—	—
Temporary neuropraxia	2 (20%)	1 (6.7%)	.543
Hematoma	1 (10%)	0	.400
Subjective dysphagia	—	1 (6.7%)	.306
Sialocele	—	—	—
Skin necrosis	—	2 (13.3%)	.500
Need for reoperation	—	—	—
Follow-up time	242.10 ± 161.88 (range: 16-455)	183.73 ± 158.290 (range: 16-576)	.345

^a^LaMiNA: Laser, Microneedling, and Nanofat.^12^

There were no significant differences in postoperative complications between cohorts. The overall complication rate was 28% (n = 7). Among all patients, 3 (12%) experienced transient neuropraxia, 1 (4.2%) developed hematoma, and 1 (4.2%) reported subjective dysphagia. All 3 patients with reported neuropraxia had transient depressor anguli oris (DAO) neuropraxia which was subsequently treated with 10 units of botulinum toxin-A for balance and was resolved within 3 weeks. The male patient in this cohort experienced DAO neuropraxia, which resolved with no sequelae. The patient with hematoma was treated conservatively with in-office needle aspiration and compression and recovered without any long-term sequalae. The patient with reported dysphagia had complete resolution of symptoms within 2 days. No seroma or sialocele formation was reported in this group. No revision surgeries were performed within a year.

### Angle Measurements

**Figure 3.**
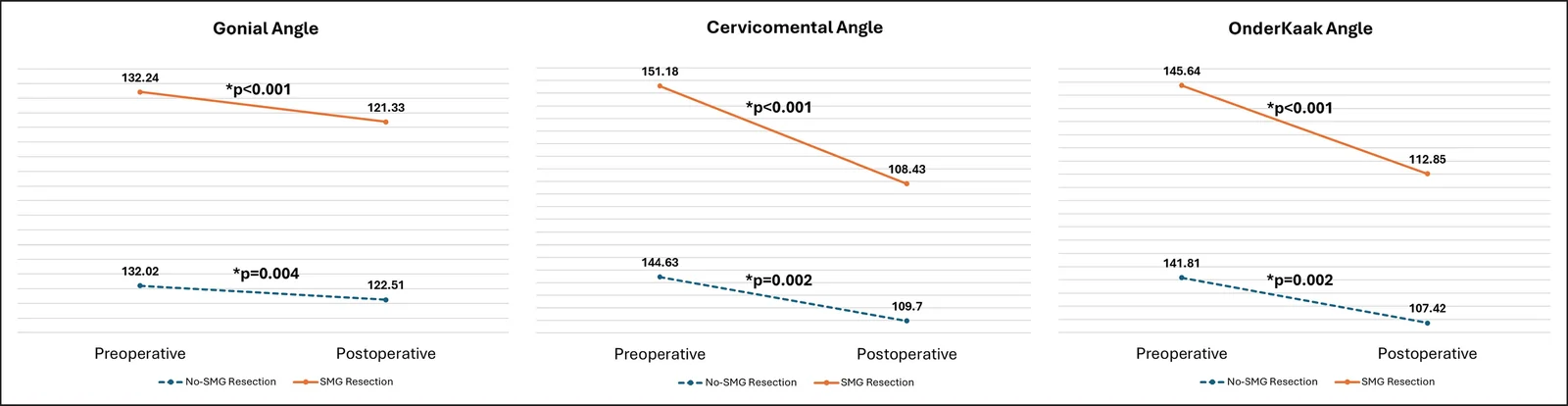
This figure shows change in preoperative and postoperative neck angle measurements (degrees°) after neck lift using the HMC neo-ligament with and without submandibular gland resection.

Figure 3 shows preoperative and postoperative neck angle measurements for each cohort. Both groups demonstrated a statistically significant reduction in angle measurements from preoperative to postoperative cervicomental (CMA), gonial (GA), and OnderKaak (OK) angles. Angle measurements were taken on average 215 days postoperatively (range: 16-576). For the no-SMG resection group, the mean preoperative CMA decreased from 144.63° ± 16.21 to 109.70° ± 16.14 (*P* = .006). The SMG resection group improved CMA from 151.18° ± 13.69 to 108.43° ± 8.06 (*P* < .001). For the no-SMG resection group, the mean preoperative GA improved from 132.02 ± 3.61 to 122.51 ± 4.32 (*P* = .004). For the SMG resection group the mean preoperative GA improved from 132.24° ± 5.20 to 121.33° ± 2.51 (*P* < .001). For the no-SMG resection group, the mean preoperative OK angle improved from 141.81° ± 17.59 to 107.42° ± 13.09 (*P* = .002). For the SMG resection group, the mean preoperative OK angle improved from 145.64° ± 16.36 to 112.85° ± 10.83 (*P* < .001).

Table 2 shows mean changes in cervicomental, gonial, and OnderKaak angles compared between patients who did not undergo SMG resection and those who did. There were no statistically significant differences in neck angle measurements between the two groups. The mean change in CMA was −34.93° ± 25.21° (range: −63.98° to 17.01°) in patients without SMG resection and −42.74° ± 15.76° (range: −70.14° to −15.82°) in the SMG resection cohort (*P* = .461). Similarly, gonial angle (GA) improvement was comparable between groups, with a mean change of −9.51° ± 4.44° (range: −14.88° to −0.79°) in the no SMG resection group and −10.90° ± 3.78° (range: −17.90° to −2.86°) in the SMG resection group (*P* = .567). Change in OnderKaak angle (OK) also demonstrated no significant difference, averaging −34.38° ± 19.93° (range: −65.03° to −6.55°) in the no SMG resection group and −32.80° ± 16.12° (range: −61.96° to −11.25°) in the SMG resection group (*P* = .849).

**Table 2.** Comparison of Mean Change in Neck Angle Measurements Between Patients With and Without Submandibular Gland Resection

Change in angle, mean ± SD (range)	No SMG resection	SMG resection	*P*-value
Cervicomental (CMA)	−34.93° ± 25.21 (range: −63.98°— 17.01°)	−42.74° ± 15.76 (range: −70.14°— −15.82°)	.461
Gonial (GA)	−9.51° ± 4.44 (range: −14.88°— 0.79°)	−10.90° ± 3.78 (range: −17.90°— −2.86°)	.567
OnderKaak (OK)	−34.38° ± 19.93 (range: −65.03°— −6.55°)	−32.80° ± 16.12 (range: −61.96°— −11.25°)	.849

**Figure 4.**
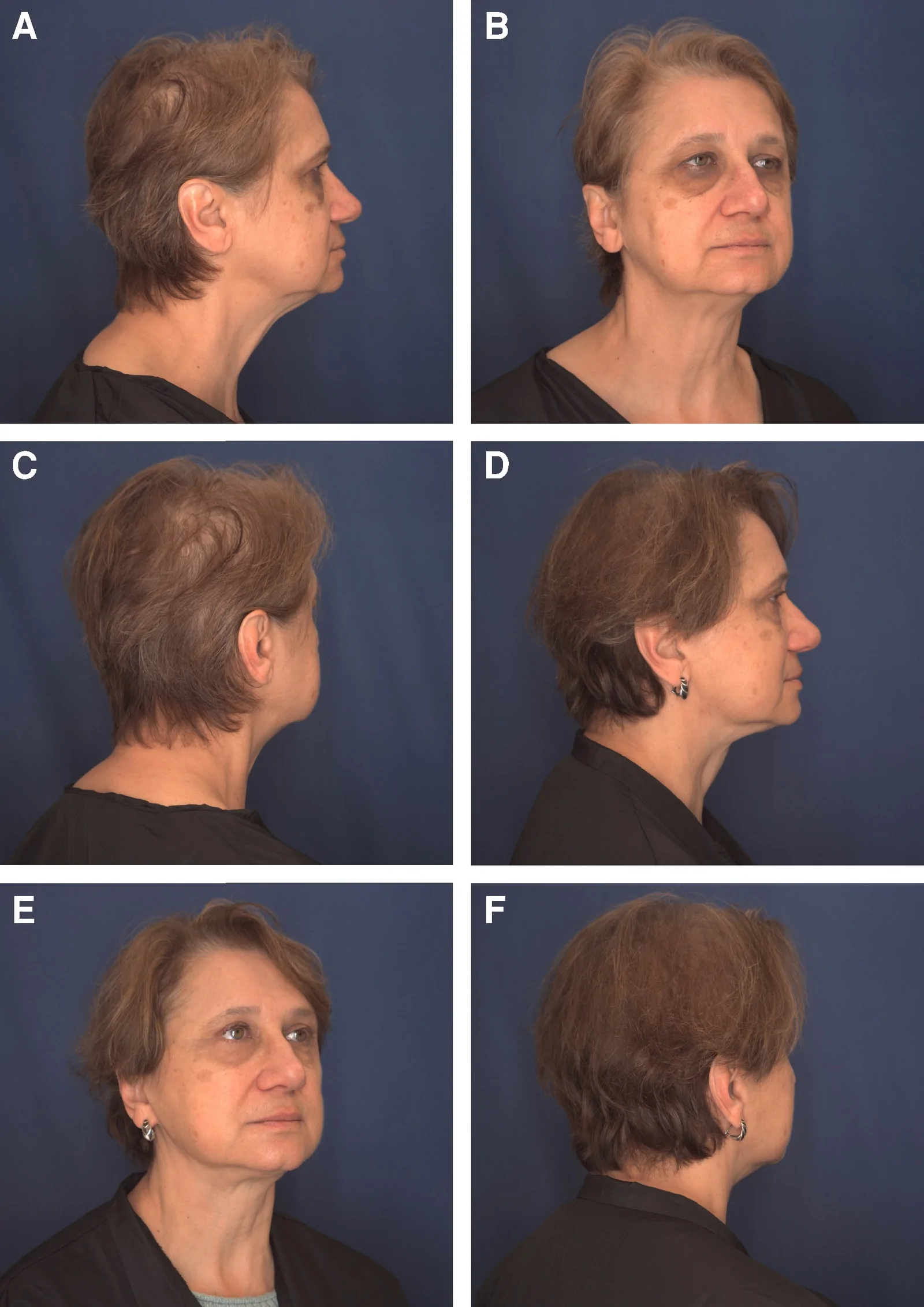
This patient is a 67-year-old female 9 months after deep plane facelift and neck lift using the HMC neo-ligament technique without submandibular gland resection. (A) Preoperative lateral view demonstrating a cervicomental angle of 159.54° and a gonial angle of 128.37°. (B) 9-month postoperative lateral view showing improvement, with a cervicomental angle of 99.27° and gonial angle of 121.16°. (C) Preoperative anterior oblique view. (D) Postoperative anterior oblique view. (E) Preoperative posterior oblique view demonstrating an OK angle measuring 157.14°. (F) Postoperative posterior oblique view demonstrating an improved OK angle measuring 115.59°.

**Figure 5.**
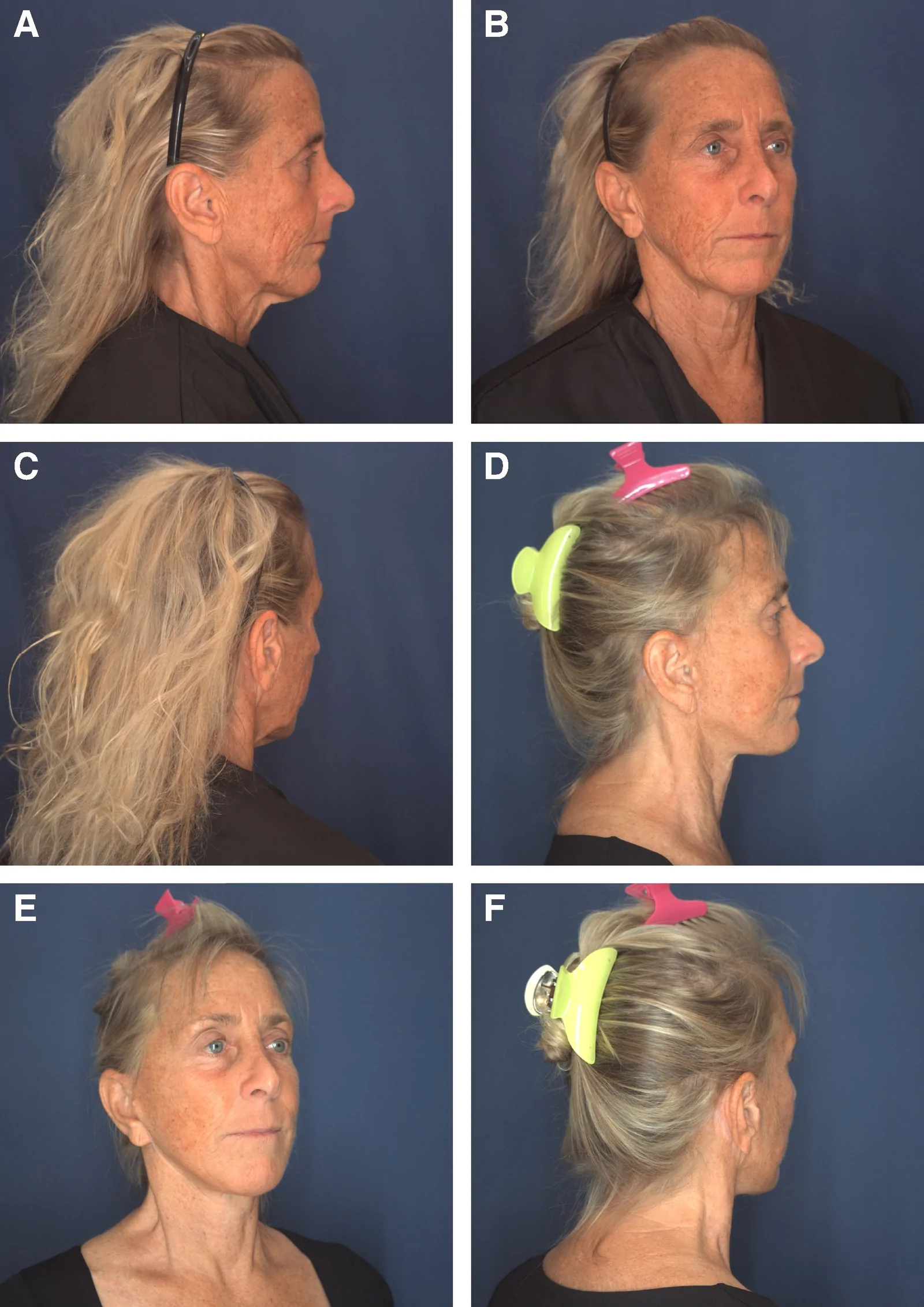
This patient is a 60-year-old female 1 year after deep plane facelift and neck lift using the HMC neo-ligament without submandibular gland resection. (A) Preoperative lateral view demonstrating a cervicomental angle of 141.59° and a gonial angle of 164.35°. (B) 1-year postoperative lateral view showing improvement, with a cervicomental angle of 99.34° and gonial angle of 151.52°. (C) Preoperative anterior oblique view. (D) Postoperative anterior oblique view. (E) Preoperative posterior oblique view demonstrating an OK angle view measuring 141.11°. (F) Postoperative posterior oblique view demonstrating an improved OK angle measuring 94.58°.

**Figure 6.**
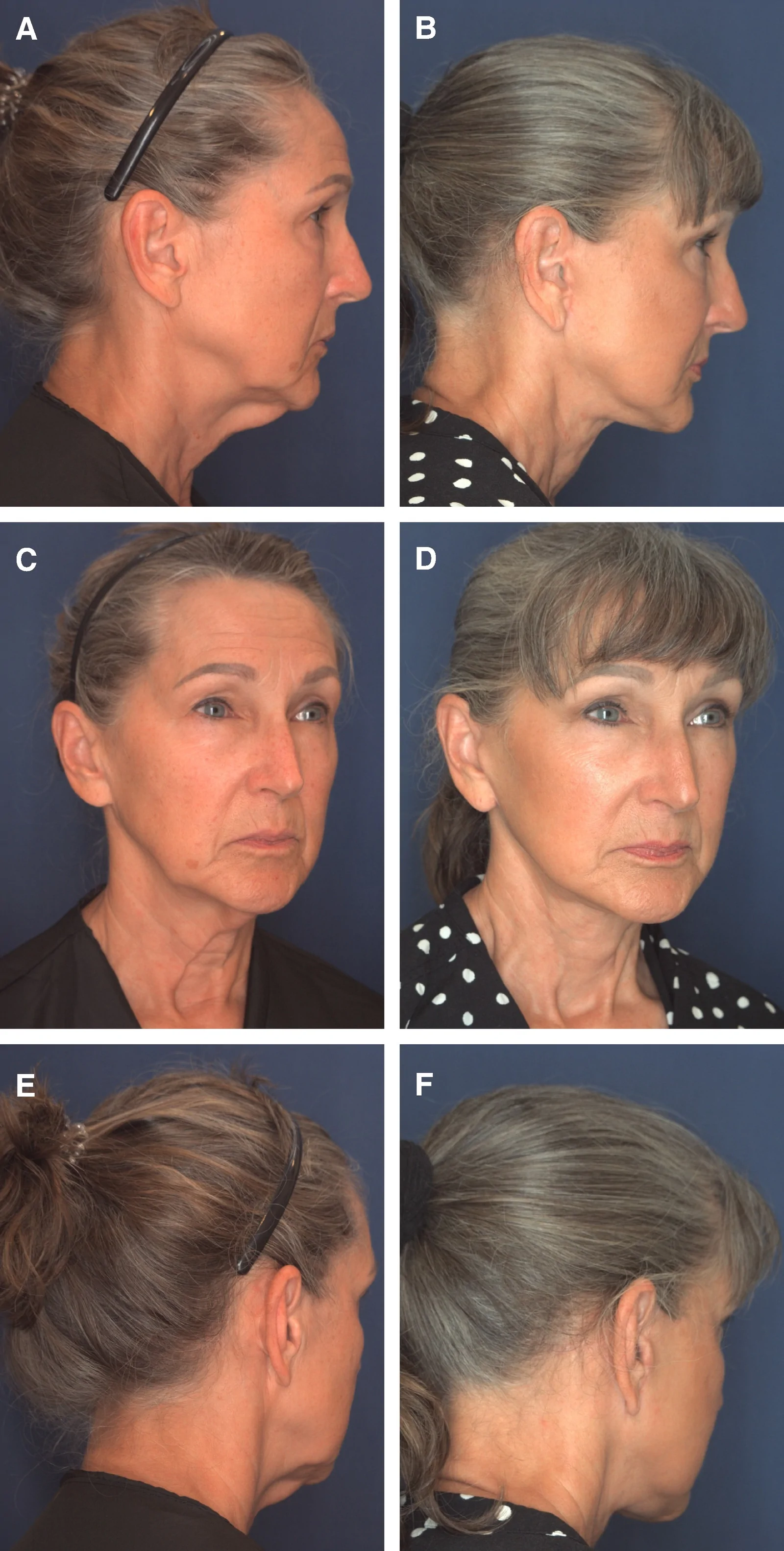
This patient is a 69-year-old female 1 year after deep plane facelift and neck lift using the HMC neo-ligament technique with partial submandibular gland resection. (A) Preoperative lateral view demonstrating a cervicomental angle of 197.47° and a gonial angle of 161.48°. **(**B) 1-year postoperative lateral view showing improvement, with a cervicomental angle of 110.6° and gonial angle of 149.29°. (C) Preoperative anterior oblique view. (D) Postoperative anterior oblique view. (E) Preoperative posterior oblique view demonstrating an OK angle view measuring 186.25°. (F) Postoperative posterior oblique view demonstrating an improved OK angle measuring 102.9°.

**Figure 7.**
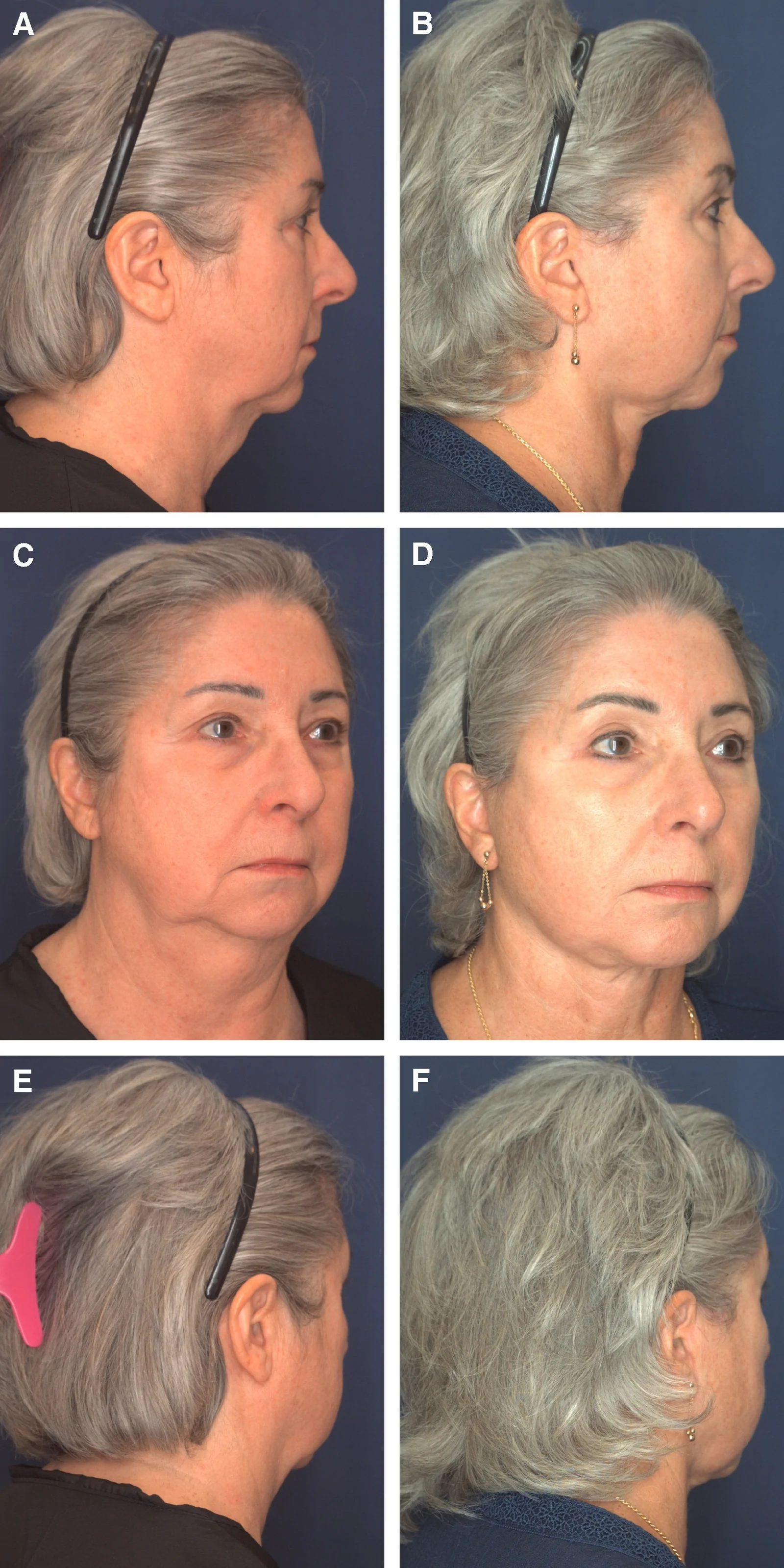
This patient is a 71-year-old female status post 6 months deep plane facelift and neck lift with HMC neo-ligament technique with partial submandibular gland resection. (A) Preoperative lateral view demonstrating a cervicomental angle of 179.34° and a gonial angle of 160.35°. (B) 1-year postoperative lateral view showing improvement, with a cervicomental angle of 129.8° and gonial angle of 120.23°. (C) Preoperative anterior oblique view. (D) Postoperative anterior oblique view. (E) Preoperative posterior oblique view demonstrating an OK angle measuring 197.8°. (F) Postoperative posterior oblique view demonstrating an improved OK angle measuring 117.30°.

Figures 4 and 5 demonstrate postoperative results using the HMC neo-ligament without submandibular gland resection. Figures 6 and 7 demonstrate postoperative results using the HMC neo-ligament with submandibular gland resection. Video 3 shows the neck in dynamic motion without any restrictions in mobility.

## DISCUSSION

This article describes the novel Hyoid-to-Mastoid Crevasse (HMC) neo-ligament, a technique in deep neck lift surgery that aims to elevate the submandibular glands and support deep neck structures through a hyoid-to-mastoid bone periosteum suspension. We also introduce the OnderKaak (OK) angle as a new aesthetic unit for evaluating the region directly overlying the SMG. This is the first article to recommend analyzing neck lift results from the posterior oblique view, also known as the reverse ogee as a more accurate representation of aesthetic jawline improvement. This preliminary cohort analysis found that the HMC neo-ligament delivered statistically significant improvements in the cervicomental, gonial, and OnderKaak angles.

Submandibular gland reduction is considered in neck lifting when physical examination reveals glandular descent contributing to neck fullness.^13-15^ Auersvald et al^16^ demonstrated in a cohort of 504 patients that submandibular gland resection should be performed to achieve optimally improved cervical contours. Similarly, Shauly et al^17^ concluded that SMG reduction is an effective adjunct to improve neck aesthetic in neck lifting. In this cohort, patients underwent SMG resection when it was determined on physical exam and intraoperatively that glandular descent was below the mandibular angle. However, some patients did not undergo SMG resection. In this study, there was no difference in the angle change between the two cohorts (SMG resection and non-SMG resection). This may suggest that the HMC neo-ligament alone may be sufficient to achieve jawline definition even in patients without submandibular gland reduction. The primary surgeon has observed that unlike previous techniques, the HMC neo-ligament effectively manages gland ptosis even when the gland is preserved. Therefore, the HMC neo-ligament provides an alternative strategy for managing gland ptosis by harnessing the tensile strength of the hyoid periosteum and mastoid periosteum to elevate and secure the SMG in a vertical-posterior direction above the level of the mandibular angle, as well as rotating the hyoid bone upward. This suspension between the two bony attachments provides a more secure suspension point for the platysma.

Interlocking suspension introduced by Giampapa represents a technique similar to the HMC neo-ligaments but with key differences. Giampapa's method consists of an interlocking suture extending from the midline to the mastoid fascia to create a loop suture at the level of the mandible to address ptotic submandibular glands, cervical lipectomy, platysmaplasty, and platysmal imbrication. While proven to increase patient satisfaction and provide long-lasting results, patients often returned complaining of persistent tightness in the submental region.^18^ This is likely as a result of excessive tension of the interlocking suture bridging the two platysmal muscle flaps. To alleviate this discomfort, additional operations were required to release one end of the suture and remove the other, resulting in a slight loss of jawline definition. The HMC neo-ligament approach addresses this issue by substituting the interlocking suture with direct anchoring to the hyoid periosteum, allowing for application of sufficient tension without the feeling of persistent tightness. This is validated by the results of our study as none of the patients in this group reported long-term excessive tension and only one patient reported subjective dysphagia, which completely resolved within 2 days.

The HMC neo-ligament is completed using a resorbable 3-0 PDS suture. An absorbable suture was intentionally selected given that this technique represents a novel approach that alters the position of the hyoid bone, thereby offering flexibility and reversibility in the event of postoperative symptoms such as dysphagia. In this cohort, one patient experienced transient, subjective dysphagia that resolved spontaneously without sequelae. As experience with the procedure grows, the primary surgeon is considering transitioning to a permanent suture; however, the current use of an absorbable suture allows for the possibility that hyoid repositioning will not be permanent, which is advantageous during the early adoption phase of this technique.

Platysmaplasty alone with plication and lateral traction to the mastoid are insufficient to adequately support a prolapsed submandibular gland. In this scenario, resection of the SMG would be indicated. However, this is not universally agreed upon and has often been a point of debate amongst plastic surgeons.^14,19^ SMG preservation is preferred when patients opt for no gland resection, or the surgeon prefers to avoid resection because of technical inexperience or to avoid complications. Understanding of the anatomy surrounding the submandibular gland is paramount to safely perform the procedure.^20^ Intraoperative bleeding during submandibular gland resection requires immediate action as it can quickly obscure the operative field, which increases the risk of neurovascular injury and airway compression.^21^ The procedure requires direct visualization of the gland and meticulous dissection techniques to avoid important vessels in this anatomic region. Due to its complexity, resecting the glands should only be done when indicated and should not be performed without extensive experience. The HMC neo-ligament technique offers an alternative option to secure the platysma and submandibular glands above the angle of the mandible by anchoring to the hyoid and mastoid periosteum. This technique delivers a secure vertical lift that may substitute partial SMG resection.

A known complication of submandibular gland resection is sialocele formation, which is a saliva filled cavity in the neck due to a disruption in the normal drainage system. It has been shown that the submandibular glands account for up to 70% of unstimulated saliva production, thus playing an essential role in providing lubricant for adequate swallowing and digestion.^22^ The literature has reported up to a 9.52% incidence of sialocele in open neck lifts.^14,23^ Santos et al^24^ attributed postoperative sialocele formation to failure to close the capsule. In the present technique, if partial gland resection is performed, the primary surgeon does not repair the capsule. As the HMC neo-ligament is passed through the underside of the platysma muscle and secured to its bony anchors, the platysma muscle applies direct compression to the partially resected submandibular gland surface and therefore recreates the capsule. One study previously showed that after partial SMG resection, the platysma can be brought in contact with the cut surface of the SMG to seal the dissected area and found a reduction in the occurrence of hematoma and sialocele.^22^ Therefore, we hypothesize that the support delivered by the HMC suture may provide sufficient pressure to the cut surface of the SMG to prevent sialocele formation, as indicated by the zero rate of sialocele formation in the study group.

The gold standard to quantify the aesthetic units of the neck are the cervicomental and gonial angles, which have been traditionally captured from a lateral view.^2^ This has been the cardinal perspective used by plastic surgeons to showcase neck lift results. However, this view inadequately evaluates the region directly overlying the submandibular glands. The primary surgeon proposes that SMG protrusion is best visualized from the reverse ogee view. Therefore, there is paucity in the available vocabulary to describe the aesthetic units of this anatomical region. The OnderKaak angle fills this crucial void in the assessment of the region below the mandible and overlying the SMG. Postoperatively, this view illustrates the high degree of jawline definition achieved by the hyoid-to-mastoid crevasse and allows surgeons to more accurately track patient progress over time and identify potential submental relapse. This study shows a statistically significant improvement in all angles from preoperative to postoperative evaluation. This may be due to the support provided by the HMC neo-ligament to anchor the platysma between the two bony structures, thus lifting the submandibular glands above the mandibular angle.

There are several limitations to consider in this study. This is a small retrospective review of a technique performed by a single-surgeon, thus definitive conclusions cannot be drawn from the outcomes. The study lacks a comparative group, and future studies are needed to analyze traditional neck lift techniques compared to the HMC neo-ligament to assess the durability and long-term benefits of this technique. Moreover, angle measurements were conducted by a single reviewer which could have introduced measurement bias. We believe that this novel technique and measurement unit add to the current literature by introducing a reproducible and versatile surgical technique for deep plane neck lifting that can be adopted by plastic surgeons early in their careers and by those already comfortable with submandibular gland resection.

## CONCLUSIONS

The HMC neo-ligament is a versatile approach to submandibular gland management in neck rejuvenation. This neo-ligament may be utilized in cases of SMG preservation or partial resection, depending on patient anatomy and preferences. When paired with OK angle, this combination offers a quantitative framework for assessing improvements in neck lift aesthetics.
