# Correction to: Redefining Neck Rejuvenation: The Novel Hyoid-to-Mastoid Crevasse Neo-Ligament and OnderKaak Angle in Deep Plane Neck Lift

**DOI:** 10.1093/asjof/ojag115

**Published:** 2026-07-01

This is a correction to: R Brannon Claytor, Patricia M Fuentes, Grace Tolan, Redefining Neck Rejuvenation: The Novel Hyoid-to-Mastoid Crevasse Neo-Ligament and Lateral Submandibular Cervical Angle in Deep Plane Neck Lift, *Aesthetic Surgery Journal Open Forum*, Volume 8, 2026, ojag009, https://doi.org/10.1093/asjof/ojag009

In the originally published version of this manuscript, the authors described the “OnderKaak angle” as a novel aesthetic unit for evaluation of the jawline and neck contour overlying the submandibular gland and presented this terminology as a new contribution to the literature. Following publication, it was identified that this region had been previously described by Bravo as the “lateral submandibular cervical angle”. Accordingly, the claim that “OnderKaak” represents a novel angle or aesthetic unit for measurement is incorrect. In the corrected version, all instances of “OnderKaak angle” or its abbreviation “OK” have been replaced with “lateral submandibular cervical” or the abbreviation “LSC.” References to the angle as a “novel” description have been removed in the abstract, and on pages 2 and 5 of the manuscript. Additionally, the missing reference to Bravo's 2018 article has been added to the reference list: Bravo FG. Reduction Neck Lift: The Importance of the Deep Structures of the Neck to the Successful Neck Lift. Clin Plast Surg. 2018 Oct;45(4):485-506. doi: 10.1016/j.cps.2018.05.002. Epub 2018 Aug 10. PMID: 30268239. The title of the manuscript has also been changed from “Redefining Neck Rejuvenation: The Novel Hyoid-to-Mastoid Crevasse Neo-Ligament and OnderKaak Angle in Deep Plane Neck Lift” to “Redefining Neck Rejuvenation: The Novel Hyoid-to-Mastoid Crevasse Neo-Ligament and Lateral Submandibular Cervical Angle in Deep Plane Neck Lift.”

